# The Effects of Self-Controlled Video Feedback on the Learning of the Basketball Set Shot

**DOI:** 10.3389/fpsyg.2012.00338

**Published:** 2012-09-11

**Authors:** Christopher Adam Aiken, Jeffrey T. Fairbrother, Phillip Guy Post

**Affiliations:** ^1^Kinesiology, Louisiana State UniversityBaton Rouge, LA, USA; ^2^Kinesiology, University of TennesseeKnoxville, TN, USA; ^3^Human Performance, Dance and Recreation, New Mexico State UniversityLas Cruces, NM, USA

**Keywords:** self-control, knowledge of performance, motor learning, knowledge of results (psychology), video self-modeling, basketball

## Abstract

Allowing learners to control some aspect of instructional support (e.g., augmented feedback) appears to facilitate motor skill acquisition. No studies, however, have examined self-controlled (SC) video feedback without the provision of additional attentional cueing. The purpose of this study was to extend previous SC research using video feedback about movement form for the basketball set shot without explicitly directing attention to specific aspects of the movement. The SC group requested video feedback of their performance following any trial during the acquisition phase. The yoked group received feedback according to a schedule created by a SC counterpart. During acquisition participants were also allowed to view written instructional cues at any time. Results revealed that the SC group had significantly higher form scores during the transfer phase and utilized the instructional cues more frequently during acquisition. Post-training questionnaire responses indicated no preference for requesting or receiving feedback following *good* trials as reported by Chiviacowsky and Wulf (2002, 2005). The nature of the task was such that participants could have assigned both positive and negative evaluations to different aspects of the movement during the same trial. Thus, the lack of preferences along with the similarity in scores for feedback and no-feedback trials may simply have reflected this complexity. Importantly, however, the results indicated that SC video feedback conferred a learning benefit without the provision of explicit additional attentional cueing.

## Introduction

Research in motor learning has demonstrated that allowing learners to control some aspect of instructional support benefits skill acquisition (for a review see Wulf, 2007). Self-control (SC) manipulations have been shown to facilitate learning for a variety of tasks, including those that require sequence learning (Chen et al., 2002; Chiviacowsky and Wulf, 2002, 2005; Patterson and Carter, 2010) and object projection (Janelle et al., 1995, 1997; Chiviacowsky et al., 2008; Kolovelonis et al., 2010). Types of previously examined SC manipulations have included physical guidance (Wulf and Toole, 1999; Wulf et al., 2001), amount of practice (Post et al., 2011), task scheduling (Keetch and Lee, 2007; Wu and Magill, 2011), video demonstration (Wrisberg and Pein, 2002; Wulf et al., 2005), and augmented feedback (Janelle et al., 1995; Chiviacowsky et al., 2008). The majority of these studies have examined the effects of SC feedback in the form of knowledge of results (KR) or, less frequently, knowledge of performance (KP).

Several explanations have been forwarded to account for SC benefits seen in motor learning research. Janelle et al. (1995, 1997) suggested that SC prompts learners to process information on a deeper cognitive level while McNevin et al. (2000) argued that SC might increase participant motivation. Another perspective forwarded by Chiviacowsky and Wulf (2002) suggested that SC allows learners to strategically tailor their experience to fit personal needs and preferences during skill acquisition. This latter argument was based on findings that SC participants reported asking for feedback after so-called *good* trials and that performance on these trials was superior to performance on no-feedback (i.e., *poor*) trials.

Another interesting aspect of SC research is that learners have typically requested instructional assistance (e.g., feedback, video demonstration, or guidance) less frequently than might be expected. For example, Wulf et al. (2005) found that SC participants requested video demonstration of a basketball jump shot on only 5.8% of acquisition trials. Similarly, Janelle et al. (1995) found that SC participants requested KR on an underhanded tossing task after approximately 7% of acquisition trials. It has also been reported that SC participants decrease requests for instructional support as practice progresses. For example, Chiviacowsky and Wulf (2002) found that KR requests were made after 44.7% of trials during the first acquisition block, but after only 28% during the last acquisition block. These findings were consistent with the idea that SC prompts deeper engagement in cognitive processes related to decisions about when instructional support is needed and how it can be strategically used to facilitate learning.

The most frequent SC manipulations have involved various types of augmented feedback, usually in the form of KR (Chiviacowsky and Wulf, 2002; Chiviacowsky et al., 2008). SC KR conditions have been shown to facilitate motor learning when compared to groups that received feedback on 100, 50, or 20% of trials, or that were yoked (YK) to the feedback schedules selected by SC counterparts (Janelle et al., 1995; Chiviacowsky and Wulf, 2002). The one study that used SC KP provided it in conjunction with verbal KP and showed that the combination facilitated learning compared to YK group, 50% KP, and 20% KP groups (Janelle et al., 1997). Typically a SC feedback group is tested against a YK group to control for a potential confound introduced by the effects of reduced frequency of feedback, which has been shown to enhance learning (Winstein and Schmidt, 1990).

Although SC feedback in general has been found to be effective for learning a variety of tasks, its use warrants further examination. One reason relates to the delivery of KP using video replay. Early research involving the use of video KP demonstrated that it facilitated the learning of complex motor skills (Baker, 1970; Rothstein and Arnold, 1976; Rikli and Smith, 1980; Van Wieringen et al., 1989; Hazen et al., 1990; Guadagnoli et al., 2002). In the SC literature, video KP in conjunction with verbal KP has also been shown to facilitate learning compared to a YK condition (Janelle et al., 1997). However, it is still unknown if video KP administered without additional verbal KP is an effective mode of feedback delivery within a SC protocol.

On the one hand, it seems reasonable to assume that the use of SC video KP would facilitate learning because previous research demonstrates SC benefits across a broad range of tasks and types of instructional support. On the other hand, some video KP research has suggested that video feedback may overwhelm novice learners with too much information, thereby reducing its instructional effectiveness (Rothstein and Arnold, 1976; Emmen et al., 1985). Because video KP conveys information about multiple aspects of performance, novice learners might not know how to effectively identify the most salient aspects of the movement to benefit learning. Rothstein and Arnold (1976) noted that the provision of attentional cues along with video replay might assist learners in effectively directing their attention to critical information in the video. Even with the addition of attentional cues, however, video KP still conveys much more information than traditional forms of feedback that typically deal with a single aspect of performance (e.g., algebraic error in meeting a time goal), which has important implications regarding reported preferences for feedback following so-called *good* trials (e.g., Chiviacowsky and Wulf, 2002).

The findings regarding the benefits of feedback after *good* trials (Chiviacowsky and Wulf, 2007) and SC participants’ preferences for feedback after such trials (Chiviacowsky and Wulf, 2002) has been based on experiments that used relatively simple laboratory-based sequential timing tasks and provided feedback on a single aspect of performance. When examining a more complex skill such as learning correct technique for a basketball set shot, one aspect of the motion might be considered *good* (e.g., correct follow-through) while another aspect might be *poor* (e.g., incorrect motion at the knee). When focusing on shooting form, the categorization of any given trial as either *good* or *poor* will likely be problematic and SC participants might find themselves in a dilemma with respect to their decisions about when to request or how to successfully use video KP. This dilemma might be remedied with the assistance of an experienced instructor (as in Janelle et al., 1997), but such support might not always be readily available. Consequently, it is important to determine if SC video KP as the sole source of feedback can facilitate motor learning.

The purpose of the present study was to examine the effects of SC video KP on the learning of basketball set shot technique by novices. Based on the SC literature, it was hypothesized that the SC group would achieve higher form scores during retention and transfer testing compared to the YK group. It was also hypothesized that the SC group would display a decreasing frequency of feedback requests as the acquisition phase progressed and that the SC group would report asking for feedback following perceived *good* trials more frequently than after *poor* trials.

## Materials and Methods

### Participants

Participants were 28 women (26.43 ± 5.23 years of age) recruited from a major city in the southeast United States. Prior to their involvement in the study, all participants read and signed an informed consent approved by the Institutional Review Board of the first author’s institution at the time of data collection. Participants were then assigned an identification number and asked to select a pseudonym to be used during the form ratings of video clips. Participants were randomly assigned to the SC (*n* = 14) and YK groups (*n* = 14). All participants were classified as novice basketball players using the criteria that they had no formal experience with organized basketball past the eighth grade.

### Apparatus and task

Data was collected in a private gymnasium using a basketball court with NCAA regulation dimensions. The basket was positioned 10 ft (3.05 m) above the court and had a rim circumference of 18 in (0.46 m). Regulation and youth free throw lines (as defined by the city recreation department) were located 15 (4.57 m) and 12 ft (3.66 m) from the backboard, respectively. The set shot task was completed using a NCAA regulation women’s basketball with a circumference of 28.5 in (0.72 m) and weight of 20 ounces (0.57 kg).

**Figure 1:**
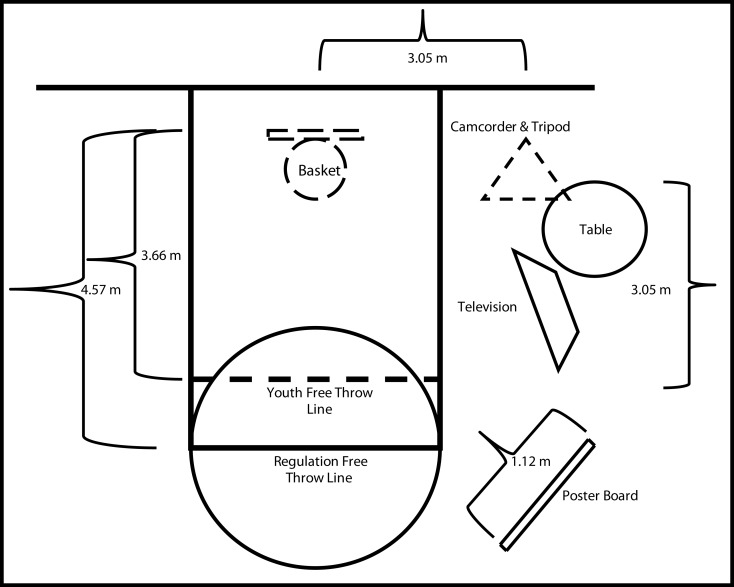
**Diagram depicting placement of equipment during the study**. (Not drawn to scale).

A video camera (Cannon ZR 960; Cannon, USA, Inc., Lake Success, NY, USA) attached to a tripod was positioned 4.57 m in front and to the right side of the participant along a 45°. The tripod height was set to 1.3 m from the bottom of the camera to best capture the whole body movement required by the task (cf. Wulf et al., 2005). The camera was connected to a 32 in (0.81 m) LCD television (LG model 32LH200C, LG Electronics, Englewood Cliffs, NJ, USA) located 3.05 m to the right of the participant and just in front of the youth free throw line. To assist participants in understanding the critical features associated with the set shot a poster (1.12 m × 0.71 m) containing the seven instructional cues for proper set shot form was located 3.05 m behind and to the right side of the participant along a 45°. The experimenter was positioned at the table next to the camera to control video playback while an assistant stood between the camera and the basket to retrieve the ball (see Figure 1).

The experimental task was a set shot used for a free throw in basketball. During acquisition and retention, participants completed the task from the youth free throw line (12 Ft). During transfer, the task was completed from the regulation free throw line (15 Ft). The primary goal of the task was to learn correct technique, but performance was assessed for both form and accuracy. For form, performance was evaluated on the first and last trial of each block during acquisition, retention, and transfer. Two skilled raters with extensive basketball experience viewed video clips in a random order and were blind to participant identity and experimental condition and phase. The raters evaluated each video clip for the presence of the seven critical features for proper set shot form and discussed the shooting form until they agreed on the score (adapted from Wulf et al., 2005). The features identified and used in the present study were: (1) proper form – stand on the line with feet shoulder width apart and toes pointed toward the basket; (2) grip/hand orientation – place shooting hand under the ball with non-shooting hand on the side for stability; (3) elbow tucked in – keep shooting arm in toward the body; (4) bend knees – bend legs so that the knees come slightly over the toes; (5) shooting motion – rapid lift of the ball to at least the forehead height with elbow under the ball pointing toward the basket simultaneous with knee extension; (6) ball release – release ball at or near the highest point; (7) follow-through – extend arm upward after ball release and flick the shooting hand (adapted from Amberry, 1996; Wulf et al., 2005; Cleary et al., 2006). For each feature, a shot was awarded a two if the feature was clearly recognizable, a one if it was somewhat recognizable, or a 0 if it was not recognizable (Wulf et al., 2005). For accuracy, the scoring system was adapted from Wulf et al. (2005) and Cleary et al. (2006). Participants were awarded points based on the following criteria:

5 points = swish (made basket, ball never touches the rim or backboard)
4 points = made basket
3 points = ball touched the rim only
2 points = ball touched both the rim and backboard
1 point = ball touched the backboard only
0 points = ball missed everything (“air ball”).

### Procedures

Participants were told that they would be using video feedback to improve their basketball shooting skills and that their goal was to improve their shooting form as much as possible. In addition, they were told not to prioritize shot accuracy at the expense of form. Participants then watched a brief (2 min, 45 s) instructional video featuring a former NCAA Division II collegiate women’s basketball player who demonstrated proper set shot technique. The video also conveyed the seven instructional features of proper set shot form. After the video, participants were informed that a list of the seven instructional cues for proper set shot form would be available to them throughout acquisition on a poster located behind them and to their right. After the participant watched the video, they took one practice shot with guidance from the experimenter and were then shown how the video feedback would be administered.

Self control participants were told that they would be allowed to access video feedback of their shooting form after any trial during acquisition. They were also told that they would not receive feedback unless they requested it. YK participants were told that they would be shown video feedback of their shooting form after some trials but not others. All participants were told that when video feedback was administered, they could watch the video as many times as they wanted (no participant watched the video for a given trial more than once). They were also told that they would not have access to video feedback or the instructional cues during retention and transfer testing.

During acquisition, participants completed 25 trials (five blocks of five trials). Each trial began with the experimenter’s assistant handing the ball to the participant who was then given a verbal cue to begin the trial. The participant was free to take as much time as needed to prepare for each shot. After the trial, the accuracy score was recorded and video feedback was administered as prescribed by the experimental condition. Data were also collected on the frequency of video feedback requests for the SC group and frequency and duration (in seconds) of poster views for both groups. Pilot testing established that a full trial was easily accomplished within 30 s, so the trials during the experiment were spaced at 30 s to equate feedback intervals with post-trial delays on no-feedback trials and to ensure that SC participants did not forego feedback in an attempt to shorten their participation. At the conclusion of each trial block, participants were given an extra 30 s break. At the end of acquisition, participants completed a post-training questionnaire about their experience receiving the video feedback. This questionnaire was similar to questionnaires used in previous research (Chiviacowsky and Wulf, 2002; Patterson and Carter, 2010). The primary difference in the questionnaire administered in the current study was that questions were expanded to a likert scale of one to five with one representing “rarely” and five representing “frequently.” Previous questionnaires asked participants about their feedback strategy but SC participants were only allowed to indicate if they requested feedback following good, bad, or both good and bad trials equally. Thus by allowing participants to answer on a likert scale, it is possible that they are able to provide more information about their feedback request strategy, which may help better understand the underlying causal mechanisms for the effect of learner-controlled feedback (see Table 1).

**Table 1:** **Mean scores from questionnaire**.

Condition	Question	*M*	SD
SC	1. Asked for feedback when I thought my form was good	2.93	1.33
	2. Asked for feedback when I thought my form was not good	3.07	0.37
YK	1. I received feedback when I needed it	3.50	1.09
	2. I received feedback after trials when my form was good	3.21	1.12
	3. I received feedback after trials when my form was not good	3.29	1.38

*SC, self-control; YK, yoked*.

*Likert scale for all questions: 1–5 (1, rarely; 3, occasionally; 5, frequently)*.

Approximately 24 h following acquisition, participants returned to the facility to complete a 10-trial retention test followed by a 10-trial transfer test. Each test consisted of two blocks of five trials each. All procedures were similar to acquisition except that no-feedback was provided, the instructional cues were not available, and trials were spaced at 15 s. Participants received a 30 s break between the end of retention and the beginning of transfer. For retention and transfer tests, shots were taken from the youth and regulation free throw lines, respectively.

### Data analyses

The primary dependent measure was form score. Shot accuracy, the number of views of the instructional cues, and the duration of viewing time when participants referred to the cues were also analyzed. For SC participants, frequency of video feedback requests was calculated for each trial block. Post-training questionnaire responses were tabulated for the SC and YK groups. For acquisition, average form scores, accuracy scores, and cue view duration were analyzed using three separate 2 (group) × 5 (block) analyses of variance (ANOVA) with repeated measures on the last factor. The number of instructional cue views by each group during the first and second halves of acquisition were compared in a 2 (group) × 2 (acquisition half) chi-square analysis. Form and accuracy scores on feedback and no-feedback trials were analyzed using two separate 2 (group) × 2 (trial type) ANOVAs with repeated measures on the last factor. For retention and transfer, form and accuracy scores were analyzed using separate 2 (group) × 2 (block) ANOVAs with repeated measures on the last factor.

## Results

### Acquisition

The overall frequency of video KP requests by the SC group was 27% throughout acquisition. The number of requests was highest during Block 1 (33%) and decreased as practice progressed through Block 5 (19%).

**Figure 2:**
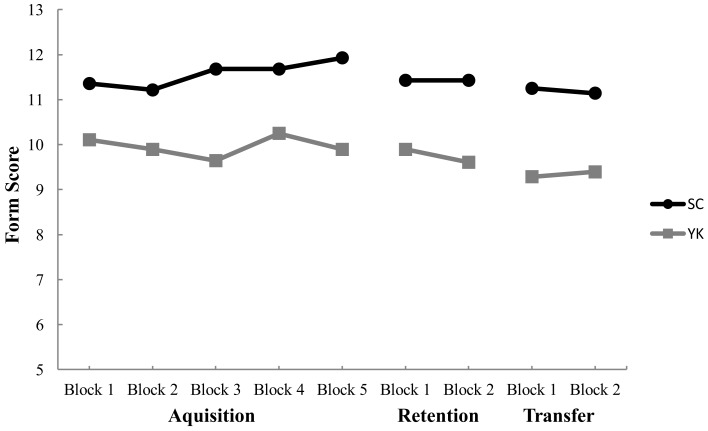
**Mean form scores for self-control (SC) and yoked (YK) groups for each trial block during acquisition, retention, and transfer**. Higher scores represent better shooting form.

Figure 2 shows mean form scores for the SC and YK groups throughout acquisition. Although the SC group produced slightly higher form scores than the YK group throughout acquisition, the differences only reach significance during Blocks 3 and 5. This observation was supported by a significant Group × Block interaction, *F* (4, 104) = 2.93, *p* = 0.042, η^2^ = 0.101. *Post hoc* testing indicated that the SC group scored significantly higher than the YK group on Block 3 (*p* = 0.034) and Block 5 (*p* = 0.023) only. There was no significant difference between groups during Blocks 1, 2, and 4. Neither the main effect for block, *F* (4, 104) = 2.27, *p* = 0.091, nor for group, *F* (1, 26) = 3.42, *p* = 0.076, were significant. Although the *post hoc* comparison for Block 1 showed no significant difference between groups (*p* = 0.177), a second examination for potential initial differences was conducted on form scores for Trial 1. The results of the *t*-test revealed no significant difference between the SC and YK groups (*p *= 0.222).

**Figure 3:**
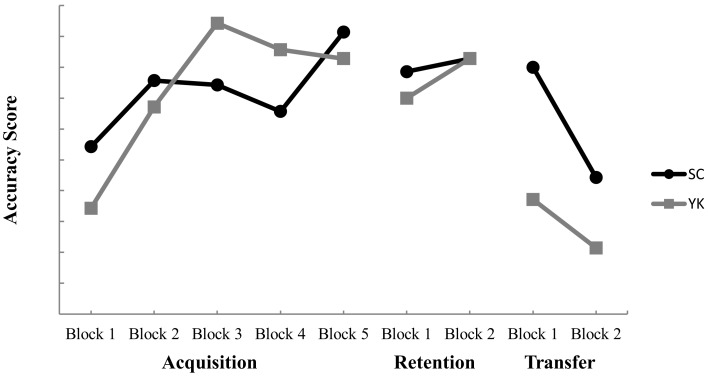
**Mean accuracy scores for self-control (SC) and yoked (YK) groups for each trial block during acquisition, retention, and transfer**. Higher scores represent more accurate performance.

Figure 3 shows mean accuracy scores for the SC and YK groups throughout acquisition. Both groups performed similarly to one another and improved accuracy across trial blocks. These observations were supported by a significant main effect for block, *F* (4, 104) = 2.60, *p* = 0.040, η^2^ = 0.091. *Post hoc* testing indicated no reliable differences between individual blocks, but the *p*-values for the comparisons between Block 1 and Blocks 3 and 5 approached the criteria for significance (*p* = 0.084 and 0.069, respectively). Neither the main effect for group, *F* (1, 26) = 0.001, *p* = 0.976, nor the Group × Block interaction, *F* (4, 104) = 0.755, *p* = 0.557, were significant.

Both groups viewed the instructional cues for a similar amount of time and the mean viewing time decreased during acquisition (Block 1, *M *= 1.9 s; Block 2, *M *= 1.75 s; Block 3, *M *= 1.2 s; Block 4, *M* = 0.7 s; Block 5, *M *= 0.7 s). The decrease in viewing time was supported by a significant main effect for block, *F* (4, 104) = 3.13, *p* = 0.036, η^2^ = 0.108. *Post hoc* analyses revealed no significant difference from one block to another, however. Neither the main effect for group, *F* (1, 26) = 2.18, *p* = 0.152, nor the Group × Block interaction, *F* (4, 104) = 0.339, *p* = 0.776, were significant. The SC group also looked at the instructional cues more frequently than the YK group. The SC group viewed the cues 34 times during the first half of acquisition and 28 times during the second half while the YK group viewed the cues 23 times and 7 times during the first and second halves of acquisition. The chi-square analysis indicated that the SC group viewed the cues more frequently than expected while the YK group viewed the cues less frequently than expected during both acquisition halves, χ^2^ = 4.09, *p* = 0.043.

Form scores on feedback and no-feedback trials were very similar for both the SC and YK groups. This observation was supported by the absence of a significant main effect for trial type, *F* (1, 24) = 1.213, *p* = 0.282, or Group × Trial Type, *F* (1, 24) = 0.258, *p* = 0.616. The main effect for group, *F* (1, 24) = 3.31, *p* = 0.082, was also not significant. In addition, accuracy scores on feedback and no-feedback trials were very similar for both groups. This observation was supported by the absence of a significant main effect for trial type, *F* (1, 26) = 0.000, *p* = 0.983, or Group × Trial Type, *F* (1, 26) = 2.69, *p* = 0.113. The main effect for group, *F* (1, 26) = 0.015, *p* = 0.903, was also not significant.

### Retention

The SC and YK groups performed similarly in terms of form scores. The main effects for group, *F* (1, 26) = 3.81, *p* = 0.062, and for block, *F* (1, 26) = 2.02, *p* = 0.167, were not significant. Neither was the Group × Block interaction, *F* (1, 26) = 2.02, *p* = 0.167. The SC and YK groups also performed similarly in terms of accuracy scores. The main effects for group, *F* (1, 26) = 0.057, *p* = 0.812, and for block, *F* (1, 26) = 0.235, *p* = 0.632, were not significant. Neither was the Group × Block interaction, *F* (1, 26) = 0.059, *p* = 0.81.

### Transfer

The SC group demonstrated higher form scores than the YK group. This observation was supported by a significant main effect for group, *F* (1, 26) = 4.67, *p* = 0.04, η^2^ = 0.153. Neither the main effect for block, *F* (1, 26) = 0.00, *p* = 1.00, nor the Group × Block interaction, *F* (1, 26) = 0.436, *p* = 0.515, were significant. In respect to accuracy scores, the SC and YK groups performed similarly during transfer. The main effects for block, *F* (1, 26) = 2.59, *p* = 0.12, and group, *F* (1, 26) = 1.57, *p* = 0.221, were not significant. Neither was the Group × Block interaction, *F* (1, 26) = 0.392, *p* = 0.537.

### Questionnaire

The average scores for the Likert scale items on the post-acquisition questionnaire are reported in Table 1. The SC group indicated that they asked for feedback *occasionally* after both *good* trials (*M *= 2.93; 3 = occasionally) and *poor* trials (*M *= 3.07). The YK group indicated that they received feedback when they needed it *occasionally* (*M *= 3.50). They also indicated that they received feedback *occasionally* after both *good* trials (*M *= 3.21) and *poor* trials (*M* = 3.29). Just under half (*n *= 6) of the YK group indicated a preference for receiving feedback after *good* trials while the others (*n* = 8) indicated a preference for feedback after *poor* trials.

The results from the open-ended questions indicated that several SC participants (*n* = 10) reported *not* asking for video KP for a number of reasons. Some (*n* = 5) chose to *not* receive feedback because their inherent feedback was as expected. That is, they felt their form was close to what was desired. Others (*n* = 4) noted that they already “knew” what they did wrong. One participant did not request feedback due to embarrassment about incorrect form. SC participants indicated using feedback when they wanted to confirm their inherent feedback about either correct or incorrect form (*n* = 8) and to evaluate their own form (*n* = 5). One participant noted that they had no specific strategy for requesting video KP.

## Discussion

The purpose of this study was to examine the effects of SC video KP on the learning of basketball set shot technique by novices. The most important contribution to motor learning research was the demonstration that SC of video KP as the sole source of feedback facilitated learning of the basketball set shot technique as evidenced by the superior form scores during transfer. This finding provided partial support for the first hypothesis and extended earlier research on the use of SC video KP. Janelle et al. (1997) previously found a SC video KP benefit when it was provided in conjunction with verbal KP from an experienced instructor. Although the inclusion of verbal KP in that study followed Rothstein and Arnold’s (1976) recommendations about using verbal cues with video feedback, it also introduced a confound that prevented a clear demonstration that SC manipulations might extend to the use of video KP, *per se*. The results of the present study indicated that SC over video KP as the sole source of feedback can benefit motor learning for a fairly complex, real-world skill.

The results regarding feedback requests supported the second hypothesis and were partially consistent with previous research (e.g., Janelle et al., 1997; Chiviacowsky and Wulf, 2002). The decreasing frequency of requests by the SC group from 33% during Block 1 to 19% during Block 5 supported the second hypothesis regarding decreasing requests. In addition, the overall frequency of requests was consistent with some previous research (Chiviacowsky and Wulf, 2002), but not as low as others (Janelle et al., 1995, 1997). A point of divergence from previous reports was the lack of differences between feedback and no-feedback trials. Chiviacowsky and Wulf (2002) reported that SC participants requested feedback after so-called *good* trials more frequently than after *poor* trials. The accuracy of the participants’ interpretations was confirmed by findings that feedback trials were more accurate than no-feedback trials. In two follow-up studies, Chiviacowsky and Wulf (2005, 2007) demonstrated that learning in a SC feedback protocol was superior when the decision to request feedback followed rather than preceded a trial and that feedback after the most accurate trials in a block facilitated learning more than feedback after inaccurate trials. Taken together, the investigations by Chiviacowsky and Wulf (2002, 2005, 2007) argue that feedback after *good* trials facilitates learning to a greater extent than feedback after *poor* trials. From this and the preference for *good* trial feedback, it appears that the benefit of SC feedback is tied to the self-evaluation of performance and a strategic decision to seek feedback when that performance is determined to be *good*. The third hypothesis of the current study (i.e., that performance on feedback trials would be superior to performance on no-feedback trials) was based on this reasoning. The current results, however, did not corroborate these earlier findings nor support the third hypothesis. Nevertheless, SC video KP did confer a learning benefit despite the lack of preferences for and distinction between *good* and *poor* trials. So the current study suggests that the mechanisms underlying SC feedback benefits may be more complex than previously believed.

The post-training questionnaire results indicated that SC participants requested feedback occasionally after both *good* and *poor* trials whereas Chiviacowsky and Wulf (2002) reported that 67% of participants indicated requesting feedback mostly after *good* trials. This discrepancy might be due to the different questionnaire formats. Chiviacowsky and Wulf’s (2002) questionnaire used items asking about when feedback was requested and included five categorically distinct response options (e.g., “mostly” after *good* trials or “mostly” after *poor* trials). The questionnaire in the current study changed the response options to a Likert scale so that participants could indicate the frequency with which they requested feedback after both *good* and *poor* trials (with responses ranging from “rarely” to “frequently”). Thus, the reported frequencies for each trial type were free to overlap, which allowed for the possibility that feedback might be requested for different reasons on different trials. Indeed, the current results indicated that SC participants requested feedback after both *good* and *poor* trials *occasionally* (which represented the middle value on the scale). This finding did not support the third hypothesis (i.e., a preference for *good* trial feedback). Interestingly, the YK participants showed a similar pattern in their *perceptions* of when they receiving feedback, indicating *occasionally* after both *good* and *poor* trials. Approximately half the YK participants reported that they would have *preferred* to receive feedback after *good* trials while the other half indicated a preference for feedback after *poor* trials. Thus, the current results produced no evidence that perceptions about the quality of a trial were systematically linked to whether or not feedback was requested (by SC participants) or received (by YK participants). Taken together, the results of the quantitative portion of the questionnaire suggested that participants did not have a clear preference for receiving feedback after *good* trials as suggested by Chiviacowsky and Wulf (2002).

The absence of a preference for receiving feedback after *good* trials might have been due to the type of feedback used in the current study. As Rothstein and Arnold (1976) pointed out, video feedback can convey large amounts of information, which could presumably deal with a wide range of form characteristics reflecting various degrees of quality. For example, a participant might have elected to view video KP because of an issue related to her follow-through, which in one case might have been executed well at the end of an otherwise *poor* trial and in another case executed poorly at the end of an otherwise *good* trial. This possibility introduces a bit of a conundrum for understanding exactly how learners might use complex feedback information such as that presented in video KP. The open-ended responses from the questionnaire indicated that participants asked for feedback to either confirm their intrinsic feedback or to evaluate their form. Both of these reasons are consistent both with traditional views of augmented feedback as providing corrective information used to guide future performance (Salmoni et al., 1984) and with Chiviacowsky and Wulf’s (2002) contention that learners use feedback to confirm successful outcomes. Moreover, the primary reason for *not* requesting feedback – that inherent feedback was as expected – could also fit either perspective. Some participants indicated that they did not request feedback because they “knew” their form was “correct” while others didn’t do so because they “knew” their form was “incorrect.” Overall, the results of the questionnaire in combination with the lack of differences between feedback and no-feedback trials indicated that the use of feedback in at least some SC settings is more complex than has been previously described.

Another finding that warrants discussion was the participants’ use of the written instructional cues provided during acquisition. Janelle et al. (1995, 1997) suggested that the benefits of SC feedback might be due to deeper information processing related to the task. Such deeper engagement in the learning process might be manifested in the number of resources that a participant uses during practice. That is, it would be expected that a more engaged learner would use more sources of information to facilitate the learning process. The finding that the SC group viewed the instructional cues more frequently than expected was consistent with this perspective and provides a plausible explanation for their superior form scores during transfer. Although the additional viewing was not associated with increased accuracy it seems reasonable to expect that success in one aspect of a task (shooting form) might enhance a learner’s motivation to continue practicing until benefits in shooting accuracy would eventually become observable.

Collectively, the results of the present study extend the possible benefits of SC feedback during skill acquisition to the use of video KP as the sole source of feedback. However, the results also suggest possible shortcomings in current explanations regarding *how* learners use feedback in SC protocols, especially when using video KP. Although it appeared that participants’ reasons for requesting feedback are tied to some form of subjective evaluation of performance, as suggested by Chiviacowsky and Wulf (2002), the present results suggested that such evaluation might not always be consistent with other measures of performance. There was no quantitative evidence to suggest that participants could distinguish between *good* and *poor* trials or that they used feedback as a way to confirm a successful performance (with respect to form). When learning a complex task such as the basketball set shot, it appears likely that learners might seek video KP for a variety of reasons related to their performance (e.g., confirmation or error correction). Chiviacowsky and Wulf (2002) argued that confirmation of success indicated the potential importance of the motivational function of augmented feedback. The present findings additionally indicate that the role(s) for augmented feedback might vary according to type of tasks being learned and the type of feedback presented.

The results of the current study have important practical and theoretical implications. From a practical standpoint, there is now evidence to support the use of SC video KP in a wider range of instructional settings. Importantly, the presence of an experienced instructor to provide attentional cueing related to video replay does not seem to be an absolute prerequisite to obtain a SC video KP benefit. Although this type of support is likely to be helpful when available, practitioners need not exclude the use of SC video KP when it is not. From a theoretical standpoint, however, it is important to note that the study design did not strictly test the independent effects of SC video feedback. The results indicated that the provision of SC prompted participants to view the instructional cues more often, and it is likely that the information in the cues also facilitated performance and learning. Although this introduces a limitation in terms of understanding the independent effects of SC video KP, the study design nevertheless represented a realistic example of how video feedback can enhance the instruction of novices. In the absence of a skilled observer to provide individualized KP, a poster with cues allows for the uniform presentation of form information to all participants. The fact that the SC group viewed the poster over twice as many times as the YK group indicated that they were engaged the learning process as suggested by Janelle et al. (1997).

The finding that participants did not indicate a preference for feedback after (or were not able to distinguish between) *good* and *poor* trials was of interest from a theoretical standpoint. Although this finding was consistent with Chiviacowsky and Wulf’s (2002) argument that SC feedback choices do not support the traditional view that feedback information is used to correct errors (Salmoni et al., 1984), neither did it support their further contention that SC participants use feedback to confirm successful performances. Consequently, the observed SC benefit in the current study is most consistent with Janelle et al.’s (1997) idea that SC enhances motivation and prompts learners to engage more deeply in learning the task. Future research should be directed at more detailed examinations of how SC participants behave when presented with feedback information on multiple aspects of task performance or via multiple modes of delivery, and should endeavor to directly assess motivation and task engagement emerging from such approaches.

## Conflict of Interest Statement

The authors declare that the research was conducted in the absence of any commercial or financial relationships that could be construed as a potential conflict of interest.

## References

[B1] Amberry T. (1996). Free Throw: Seven Steps to Success at the Free Throw Line. New York, NY: Harper Perennial

[B2] Baker H. P. (1970). Film and Videotape Feedback: A Review of Literature. Washington, DC: Bureau of Education

[B3] Chen D. D. Hendrick J. L. Lidor R. (2002). Enhancing self-controlled learning environments: the use of self-regulated feedback information. J. Hum. Mov. Stud. 43, 69–86

[B4] Chiviacowsky S. Wulf G. (2002). Self-controlled feedback: does it enhance learning because performers get feedback when they need it? Res. Q. Exerc. Sport 73, 408–415 12495242 10.1080/02701367.2002.10609040

[B5] Chiviacowsky S. Wulf G. (2005). Self-controlled feedback is effective if it is based on the learner’s performance. Res. Q. Exerc. Sport 76, 42–48 10.5641/027013605X13076330976713 15810769

[B6] Chiviacowsky S. Wulf G. (2007). Feedback after good trials enhances learning. Res. Q. Exerc. Sport 78, 40–47 10.5641/193250307X13082490460346 17479573

[B7] Chiviacowsky S. Wulf G. Medeiros F. L. Kaefer A. Tani G. (2008). Learning benefits of self-controlled knowledge of results in 10-year old children. Res. Q. Exerc. Sport 79, 405–410 10.5641/193250308X13086832906238 18816953

[B8] Cleary T. J. Zimmerman B. J. Keating T. (2006). Training physical education students to self regulate during basketball free throw practice. Res. Q. Exerc. Sport 77, 251–262 10.5641/027013606X13080769704640 16898280

[B9] Emmen H. Wesseling L. Bootsma R. Whiting H. Van Wieringen P. (1985). The effects of video-modeling and video-feedback on the learning of the tennis serve by novices. J. Sports Sci. 3, 127–138 10.1080/02640418508729742 4094023

[B10] Guadagnoli M. Holcomb W. Davis M. (2002). The efficacy of video feedback for learning the golf swing. J. Sports Sci. 20, 615–622 10.1080/026404102320183176 12190281

[B11] Hazen A. Johnstone C. Martin G. L. Srikameswaran S. (1990). A videotaping feedback package for improving skills of youth competitive swimmers. Sport Psychol. 4, 3, 213–227.

[B12] Janelle C. M. Barba D. A. Frehlich S. G. Tennant L. K. Cauraugh H. (1997). Maximizing performance feedback effectiveness through videotape replay and a self-controlled learning environment. Res. Q. Exerc. Sport 68, 269–279 9421839 10.1080/02701367.1997.10608008

[B13] Janelle C. M. Kim J. Singer R. N. (1995). Subject-controlled performance feedback and learning of a closed motor skill. Percept. Mot. Skills 81, 627–634 10.2466/pms.1995.81.2.627 8570369

[B14] Keetch K. M. Lee T. D. (2007). The effect of self-regulated and experimenter imposed practice schedules on motor learning for tasks of varying difficulty. Res. Q. Exerc. Sport 78, 476–486 10.5641/193250307X13082512817543 18274219

[B15] Kolovelonis A. Goudas M. Dermitzaki I. (2010). Self-regulated learning of a motor skill through emulation and self-control levels in physical education setting. J. Appl. Sport Psychol. 22, 198–212 10.1080/10413201003664681

[B16] McNevin N. Wulf G. Carlson C. (2000). Effects of attentional focus, self-control, and dyad training on motor learning: implications for physical rehabilitation. Phys. Ther. 80, 373–385 10758522 10.1093/ptj/80.4.373

[B17] Patterson J. Carter C. (2010). Learner regulated knowledge or results during the acquisition of multiple timing goals. Hum. Mov. Sci. 29, 214–227 10.1016/j.humov.2009.12.003 20338655

[B18] Post P. G. Fairbrother J. T. Barros J. A. C. (2011). Self-controlled amount of practice benefits learning of a motor skill. Res. Q. Exerc. Sport 83, 474–481 21957706 10.1080/02701367.2011.10599780

[B19] Rikli R. Smith G. (1980). Videotape feedback effects on tennis serving form. Percept. Mot. Skills 50, 895–901 10.2466/pms.1980.50.3.895

[B20] Rothstein A. L. Arnold R. (1976). Bridging the gap: application of research on videotape feedback and bowling. Mot. Skills Theory Pract. 1, 35–64

[B21] Salmoni A. Schmidt R. A. Walter C. B. (1984). Knowledge of results and motor learning: a review and critical reappraisal. Psychol. Bull. 95, 355–386 10.1037/0033-2909.95.3.355 6399752

[B22] Van Wieringen P. C. W. Emmen H. H. Bootsma R. J. Hoogesteger M. Whiting H. T. A. (1989). The effects of video-feedback on the learning of the tennis service by intermediate players. J. Sports Sci. 7, 153–162 10.1080/02640418908729833 2810468

[B23] Winstein C. J. Schmidt R. A. (1990). Reduced frequency of knowledge or results enhances motor learning. J. Exp. Psychol. Learn Mem. Cogn. 16, 677–691 10.1037/0278-7393.16.4.677

[B24] Wrisberg C. A. Pein R. L. (2002). Note on learners’ control of the frequency of model presentation during skill acquisition. Percept. Mot. Skills 94, 792–794 10.2466/PMS.94.2.792-794 12081283

[B25] Wu W. F. W. Magill R. A. (2011). Allowing learners to choose: self-controlled practice schedules for learning multiple movement patterns. Res. Q. Exerc. Sport 83, 449–457 21957703 10.1080/02701367.2011.10599777

[B26] Wulf G. (2007). Self-controlled practice enhances motor learning: Implications for physiotherapy. Physiotherapy 93, 96–101 10.1016/j.physio.2006.08.005

[B27] Wulf G. Clauss A. Shea C. H. Whitacre C. A. (2001). Benefits of self-control in dyad practice. Res. Q. Exerc. Sport 72, 299–303 11561396 10.1080/02701367.2001.10608964

[B28] Wulf G. Raupach M. Pfeiffer F. (2005). Self-controlled observational practice enhances learning. Res. Q. Exerc. Sport 76, 1, 107–111. 10.5641/027013605X13076330976948 15810775

[B29] Wulf G. Toole T. (1999). Physical assistance devices in complex motor skill learning: Benefits of a self-controlled practice schedule. Res. Q. Exerc. Sport 70, 265–272 10522284 10.1080/02701367.1999.10608045

